# Hydraulic performance of a pre-aerated stilling basin: Experimental study

**DOI:** 10.1371/journal.pone.0318191

**Published:** 2025-01-24

**Authors:** Yu Zhou, Tengzhi Huang, Ke Xu, Jiakai Mei, Siwei Jia, Qiaoni Yu, Jinglin Qian

**Affiliations:** 1 Nanxun Innovation Institute, Zhejiang University of Water Resources and Electric Power, Hangzhou, Zhejiang, China; 2 Key Laboratory for Technology in Rural Water Management of Zhejiang Province, Zhejiang University of Water Resources and Electric Power, Hangzhou, Zhejiang, China; 3 School of Hydraulic Engineering, Zhejiang University of Water Resources and Electric Power, Hangzhou, Zhejiang, China; 4 Zhejiang WaterFocus Engineering Consulting Company, Hangzhou, Zhejiang, Jilin, China; 5 School of Hydraulic Engineering, Changchun Institute of Technology, Changchun, China; 6 Yiwu Andy Water Conservancy and Hydropower Survey and Design Co., Ltd., Jinhua, Zhwjiang, China; Ural Federal University, Ural Power Engineering Institute, RUSSIAN FEDERATION

## Abstract

Spillway chutes are critical in dam flood control, particularly in high dams where high water heads and large discharge in narrow canyons amplify the demand for safe discharging. For large unit discharges in spillways, aeration protection is essential to prevent cavitation erosion, but challenges arise from air duct choking in the traditional spillway and nonaerated regions in the stepped spillway. This paper introduces a novel spillway called the pre-aerated stilling basin spillway (PSBS). The primary distinction between PSBS and conventional spillways lies in the placement of aeration facilities. Conventional spillways feature a curved transition section that directly connects to the spillway. In contrast, the PSBS is equipped with a WES crest weir and a vertical sill of the same width, which converts supercritical to subcritical flow. This process strongly entrains air through hydraulic jumps at spillway entrances, thereby preventing cavitation damage. The study aims to experimentally investigate the flow regime, energy dissipation within the PSB, and the near-wall air concentration to ensure effective mitigation of cavitation damage downstream. The findings highlight the impact of the PSB on changing the jump type and relative local energy loss, influenced by the incoming Froude number, sill height, and sill length. Influenced by these factors, the variation in air concentration is crucial for reducing cavitation damage, necessitating potential adjustments in prototype applications to account for scale effects. Under such a pre-aeration approach, the air concentration along the downstream spillway warrants further investigation for the continuous optimization of the PSB’s design and its integration with spillways.

## Introduction

With the development of hydropower engineering, the heights of some dams have reached and even exceeded 300 m. Such high dams exhibit the traits of high water heads, large flow discharge, and narrow canyons. Therefore, the spillways with sidewall constrain designed to release excess floodwaters must contend with conditions of high unit discharge. To ensure the safe flow of high unit discharge, spillway chutes necessitate the integration of aeration devices, consisting of a ramp, a step, and a groove, to mitigate cavitation erosion [1,2]. However, if the spillway chute has a small slope, the flow rollers rear of the air entrainment cavity retrogress and cause air duct choking for a large unit discharge, resulting in dangerous local cavitation damage. This makes it difficult to achieve adequate aeration protection using typical chute aerators (e.g., deflector or offset).

In spillway chutes characterized by macro-roughness (e.g., stepped spillway), they demonstrate superior anti-cavitation performance compared to traditional spillway chutes, which is attributed to their elevated aeration concentration. With the development of roller-compacted concrete (RCC) technology, as well as other construction techniques, the application of stepped chute has become increasingly prevalent [3,4]. Generally, the flow over the stepped spillway may be either nappe, transition, or skimming flow [5]. The skimming flow is characterized by the appearance of a nonaerated region between the first step and the inception point of air entrainment. With an increase in the unit discharge, the inception point of air entrainment continuously moves downstream, resulting in an extended nonaerated region [6]. Due to the negative pressure on the vertical step face, traditional stepped spillways encounter issues such as cavitation risk when the unit discharge exceeds 30 m^2^/s [7]. In addressing this issue, two prevailing solutions are discernible. The first solution is the modification of the steps itself by integrating diverse elements, augmenting its roughness (as seen in steps with baffle, sill, or pool wall), enhancing the shear interactions between the steps and the water flow, even to the point of inducing hydraulic jumps within the step edges, thus amplifying aeration effects. The alternative approach involves the incorporation of aeration apparatuses upstream of the stepped spillway, such as pre-aerated stepped spillways. This technique introduces air into the flow, advancing the inception point of air entrainment and thereby minimizing or obviating the nonaerated region. Pfister et al. [8] and Terrier et al. [9] mounted an aerator upstream of the first step of the stepped chute, and Zamora et al. [10] added an aerator at the first vertical step face. Wang et al. [11] and Zheng et al. [12] proposed a united energy dissipation system by setting aerators such as flaring gate piers or aerated piers upstream of the stepped chute. Their aeration facilities were efficient at introducing air into the flow by deflecting the bottom and laterally of a jet. However, the jet flow may create scour and may place the spillway chute and/or the dam at risk for failure [13].

**Fig 1:**
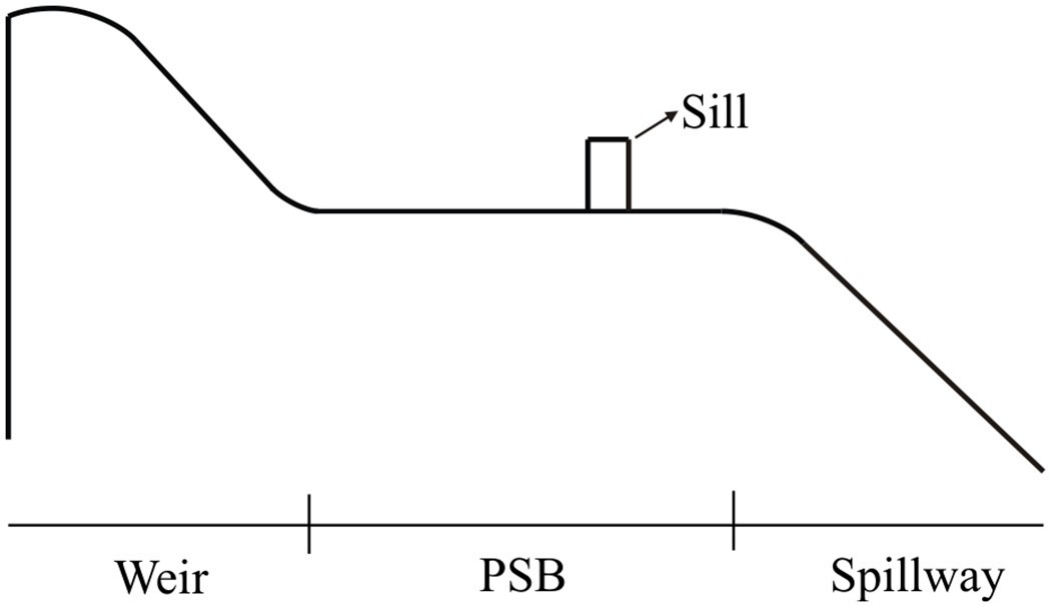
Sketch of the pre-aerated stilling basin spillway.

With regard to large unit discharge conditions for spillway chutes, it is crucial to mitigate cavitation erosion issues while striving to maintain a stable flow regime and effectively enhance energy dissipation. In this study, a new type of spillway chute was first proposed called pre-aerated stilling basin spillway (PSBS), which aims to produce aerated flow using hydraulic jump to prevent cavitation damage and enhance energy dissipation. This concept is especially pertinent to the Zhejiang Kaihua Reservoir Project in China, where comparable flow conditions are observed in its spillway design, and where this PSBS also serves as a reference for the design. The primary distinction between PSBS and conventional spillways lies in the aeration facilities, which are situated at the spillway’s transition section, in contrast to conventional spillways where the transition section, featuring a curve, directly connects to the spillway. As illustrated in Fig 1, each part of this structure includes a WES crest weir, a pre-aerated stilling basin with a sill (PSB), and a spillway. On one hand, the part, i.e., PSB, similar to stilling basin with baffles or blocks, can induce a hydraulic jump under design flow conditions. Additionally, the length of the PSB should be minimized to reduce the construction cost. A hydraulic jump may occur in the PSB by converting the supercritical to subcritical flow regimes. Because the hydraulic jump is characterized by strong air entrainment in the jump roller [14,15]. Over the past decades, researchers such as Hager and Li [16], Hornung et al. [17], Mossa [18], and Zhou et al. [19] have extensively investigated the hydraulic jump, scrutinizing the intricacies of its formation, characteristics, and particularly the mechanisms behind air entrainment that contribute to its internal aeration effect.

Overall, the design of this novel structure primarily aims to examine the flow regime, energy dissipation, and near-wall air concentration at the spillway entrance for the PSB, to ascertain whether the aerated outflow can effectively mitigate cavitation damage for the downstream spillway. As illustrated in Fig 1, diverging from conventional stilling basins, the PSB is shorter and directly connected to a downstream spillway with a given slope (even a horizontal plain), resulting in the jump type with no downstream tailwater. Owing to the special tailwater condition, an undulating wave type flow may occur in the PSB, and the downstream flow is characterized by supercritical flow conditions. This paper aims to theoretically and experimentally investigate the hydraulic characteristics of the PSB, including its flow regimes, the outflow aeration effect (the air concentration on the bottom and sidewall), and their relationships. Relationships developed from this research are applicable to the geometric design of the PSB.

## Dimensionless analysis

**Fig 2:**
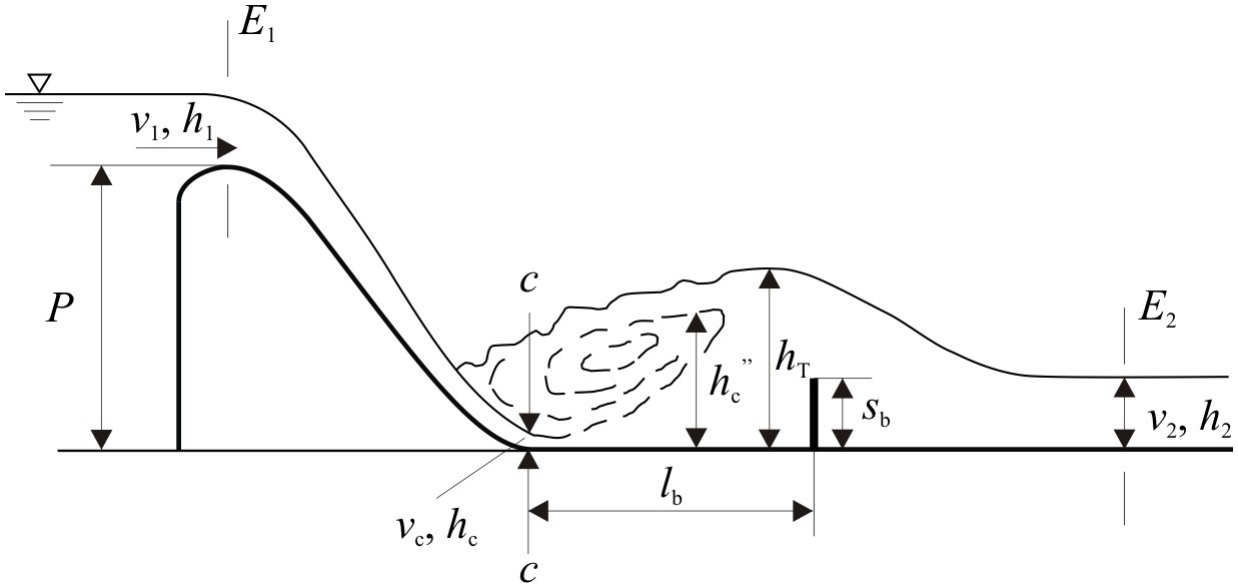
Sketch of flow through the PSB.

To better understand and provide guidance toward the flow regimes and outflow aeration effect of the PSB, a simplified model referring to the PSB was established, including a WES crest weir and a sill (Fig 2). Preliminary experiments have demonstrated that due to the water-blocking effect of the sill and the free flow conditions downstream, the water surface in the vicinity of the sill first rises and then falls [20]. In this figure, *h*_1_ and *v*_1_ is the upstream flow depth and velocity at the crest section, *h*_2_ and *v*_2_ is the flow depth downstream of PSB when the flow is stable, *l*_b_ and *s*_b_ is the sill length and height of the PSB, *P* is the weir height, *h*_T_ is the maximum flow depth, and *h*_c_^”^ is the conjugate depth of the flow depth *h*_c_ at the contraction section. *E*_1_ and *E*_2_ is the total head upstream and downstream of the PSB.

The flow passing through the PSB theoretically depended on the hydraulic and geometric parameters in the PSB. According to Buckingham’s method of dimensional analysis, the functional relationship describing the flow regimes (*FR*), outflow aeration effect (*AE*) and energy loss (Δ*H*) of the PSB could be expressed as

$$FR,AE,\Delta H=f\left(h_{1},v_{1},P,s_{b},l_{b},g,\mu ,\rho \right)$$ (1)

Considering *h*_1_, *g*, and *μ* as independent variables, Eq (1) could be constructed in view of dimensional analysis as

$$FR,AE,\frac{\Delta H}{h_{1}}=f\left(Fr_{\text{1}},\frac{P}{h_{\text{1}}},\frac{s_{b}}{h_{\text{1}}},\frac{l_{b}}{h_{\text{1}}},\text{Re}\right)$$ (2)

where *Fr*_1_ = *v*_1_/(*gh*_1_)^0.5^ is the upstream Froude number; *P*/*h*_1_ is the dimensionless weir height; *s*_b_/*h*_1_ and *l*_b_/*h*_1_ is the dimensionless sill height and length of the basin, respectively; *Re* is the Reynolds number. In the study by Hager & Bremen [21], for air-water flow, when the Reynolds number (*Re*) exceeds 10^5^, viscous effects can be neglected. Therefore, within the Reynolds number range considered in this study, prototype conditions can be adequately represented. Substituting the Froude number *Fr*_c_ at the contraction section (section c-c in Fig 2) into *Fr*_1_ in Eq (2) by using continuity equation, and weir height *P* in this study is equal to a constant. Eq (2) reduces to

$$FR,AE,\frac{\Delta H}{h_{1}}=f\left(Fr_{c},\frac{s_{b}}{h_{\text{1}}},\frac{l_{b}}{h_{1}}\right)$$ (3)

For the PSB, the flow regimes could be described in terms of hydraulic jumps. It is known that the hydraulic jump can be classified as a free, repelled, or submerged jump according to the jump location referring to the contraction section. Different jump types are associated with different submergence degree *σ*, which is the ratio of *h*_T_ to *h*_c_^”^. *σ* = 1, 0 < *σ* < 1, and *σ* > 1, for the free, repelled, and submerged jump, respectively [22,23]. Once the flow over the spillway chute becomes aerated, the air concentration downstream is larger to prevent cavitation damage [24]. The air concentration of the outflow (*C*) of the PSB is a crucial parameter determining the air concentration over the spillway chute. Furthermore, the energy dissipation effect due to the installation of the end sill can be expressed by the relative local energy loss (Δ*H*/*h*_1_), where Δ*H = E*_1_–*E*_2_.

Thus, the flow regimes, pre-aeration effect and energy dissipation of the PSB could be described by the submergence degree (*σ*), air concentration of the outflow (*C*) and relative local energy loss (Δ*H*/*h*_1_). Eq (3) could be rewritten as

$$\sigma ,C,\frac{\Delta H}{h_{1}}=f\left(Fr_{c},\frac{s_{b}}{h_{\text{1}}},\frac{l_{b}}{h_{1}}\right)$$ (4)

The experimental cases were designed according to Eq (4).

## Experimental setup and methodology

### Experimental setup

**Fig 3:**
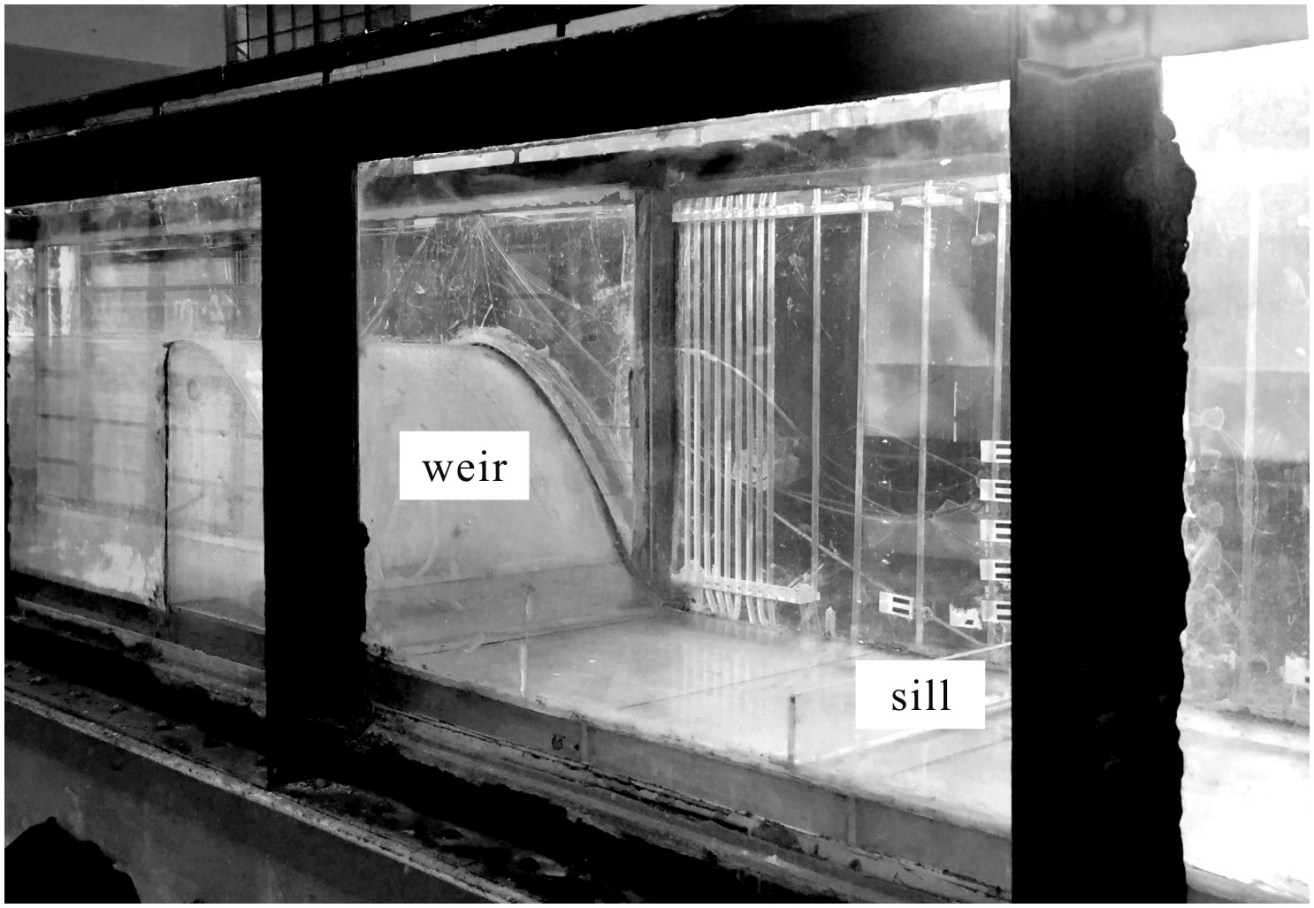
Experimental set-up.

Fig 3 shows the experimental setup, which consisted of a large feeding basin, a pump, an approach conduit, a rectangular flume, a test model, and a flow return system with electric discharge measurement. The rectangular flume made of Perspex was 25.00 m in length, 0.50 m in width, and 0.60 m in height. The test model comprised a standard Waterways Experiment Station (WES) weir and a sill, performed at a 1:70 scale. The weir crest profile was defined by the equation of *y* = 1.81*x*^1.85^ and a chute with an angle of 57° to the basin bottom. The weir height *P* was 0.36 m. The sill was set to 1.0 cm in consideration of neglecting its thickness, and its width was equal to the flume. The sill position, based on the design head conditions, places it at a distance from the weir toe that is approximately half the length of a Type I stilling basin. The sill position and height (i.e., experimental cases), as well as the experimental flow conditions, such as the inflow unit discharge (*q*), the Froude number at the contraction section (*Fr*_c_) and Reynolds number (*Re*), were listed in Table 1. Cases M12, M22, and M32 served as the effect of *l*_b_, while Cases M31, M32, and M33 were for the effect of *s*_b_.

**Table 1:** Experimental cases.

Cases	*l*_b_ (cm)	*s*_b_ (cm)	Remarks
**M12**	**50**	**6.5**	**M12-M32, effect of *l***_**b**_**, M31-M33, effect of *s***_**b**_**, 3.43 ≤ *Fr***_**c**_ **≤ 4.85, 3.97×10**^**5**^ **≤ *Re* ≤ 9.14×10**^**5**^
**M22**	**60**	**6.5**	
**M32**	**70**	**6.5**	
**M31**	**70**	**5.0**	
**M33**	**70**	**8.0**	

### Experimental methodology

All experimental cases were carried out in discharges *Q* ranging between 0.051 m^3^/s and 0.115 m^3^/s, measured via a permanent magnetic flowmeter with a precision of ±0.002 m^3^/s. Based on the model scale, the corresponding unit discharge in the prototype ranges from 60 m^2^/s to 134.7 m^2^/s, representing relatively large unit discharge conditions under spillway operations. Because the strong air-water interaction phenomenon is observed in the vicinity of the jump occurrence location [25–27], the flow depth (*h*_c_) and velocity (*v*_c_) at the contraction section are calculated from

$$h_{c}=E_{1}-\frac{q^{2}}{2g\varphi^{2}h_{c}^{2}}$$ (5)

$$v_{c}=\frac{q}{h_{c}}$$ (6)

where *E*_1_ is the total head at the weir crest section, *g* is the acceleration of gravity, and *φ* is the coefficient of velocity (*φ* = 0.95 for this weir type). The resultant *Fr*_c_ is calculated by *Fr*_c_ = *v*_c_/(*gh*_c_)^0.5^, which varied from 4.85 to 3.43 in this study. The submergence degree *σ* is the ratio of *h*_T_ to *h*_c_^”^, where *h*_c_^”^ is generally obtained by

$$h_{c}^{\prime \prime }=\frac{1}{2}h_{c}\left(\sqrt{1+8\frac{q^{2}}{gh_{c}^{3}}}-1\right)$$ (7)

**Fig 4:**
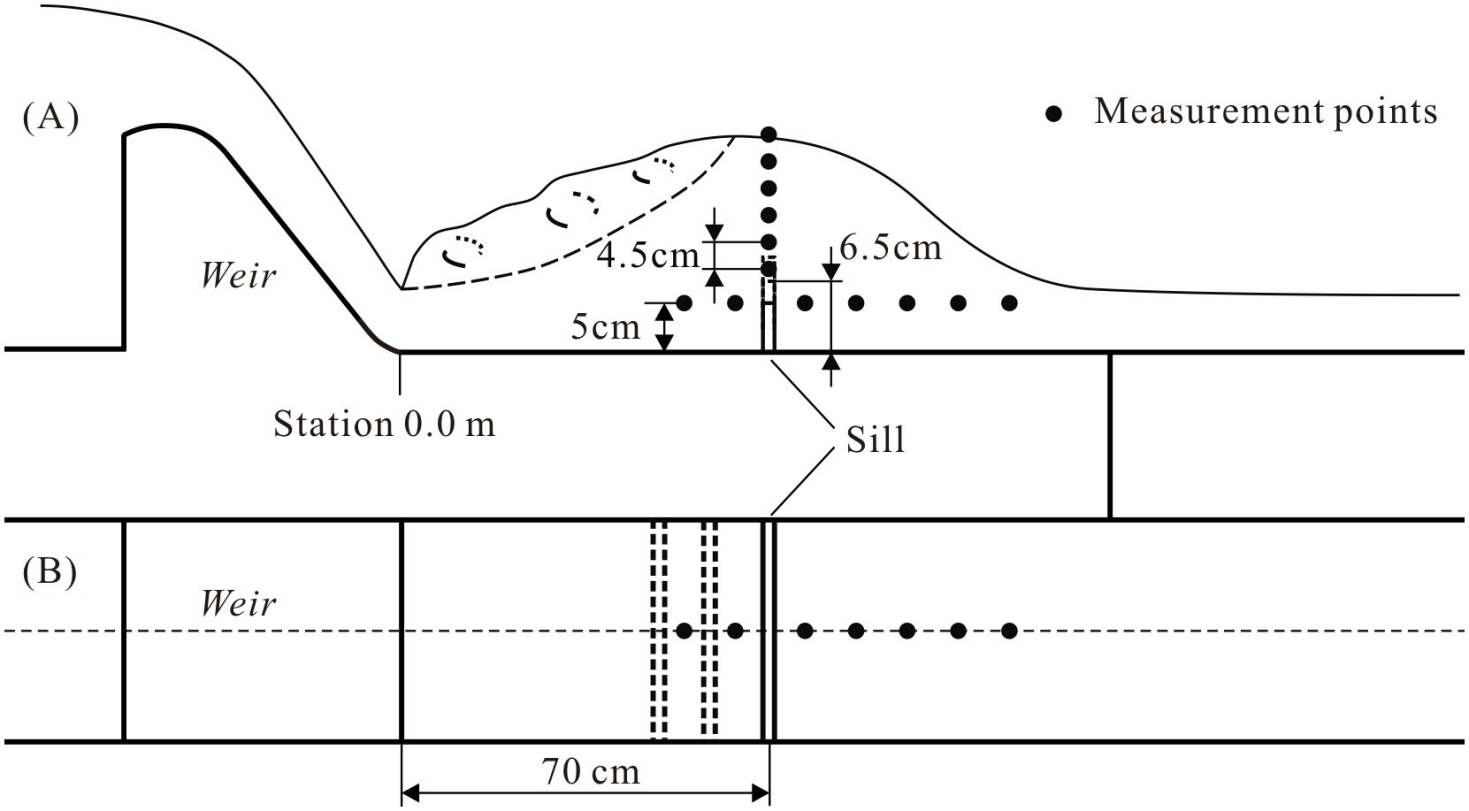
The layout of the air concentration measurement points. (A) Main view. (B) Plain view.

The relevant parameters of *h*_1_ and *h*_T_ were measured by a point gauge of 1 mm reading accuracy. The establishment of the aeration device, i.e., PSB, aims to provide aerated flow to the downstream while minimizing its footprint. Additionally, due to deaeration occurring in the hydraulic jump, this paper primarily focuses on the changes in aeration concentration in the localized areas of the bottom and sidewalls. The layout of the air concentration measurement points is shown in Fig 4. Considering that the air concentration sensor has a width of 3 cm, a pair of parallel electrodes on the sensor are utilized to measure the electrical resistance between them. The center line between the two electrodes serves as the measurement point for air concentration, and their arrangement is aligned with the direction of flow. For the measurement point at the sill position, there were six measurement points above the bottom, located from the lowest to the highest at 7.0 cm and 29.5 cm for every 4.5 cm interval, respectively.

For measurement points downstream of the sill, these points were installed both along the centerline on the bottom and 0.05 m above the bottom on the sidewall at the same Station, which corresponded to Station 0.55 m, 0.65 m, …, and 1.05 m (from the first to the last for every 0.1-m interval), respectively. The air concentration both near the bottom and the sidewall were measured using a CQ6-2005 aeration apparatus, with a sampling rate of 1020 Hz over a 30-second duration and an accuracy of ±0.3%. The CQ6-2005, made by the China Institute of Water Resources and Hydropower Research (Beijing), is a resistance-type of instrument that collects and processes the aeration data on walls by sensors (which contain a pair of parallel electrodes) and a microcomputer [28]. The principle of measurement is to convert the electric resistance values obtained from calibrated clear water and air, corresponding to air concentrations of 0 and 1, respectively. By measuring the electric resistance of the air-water flow, the corresponding air concentration can then be calculated.

## Results and discussions

### Flow regimes

#### Flow observation

**Fig 5:**
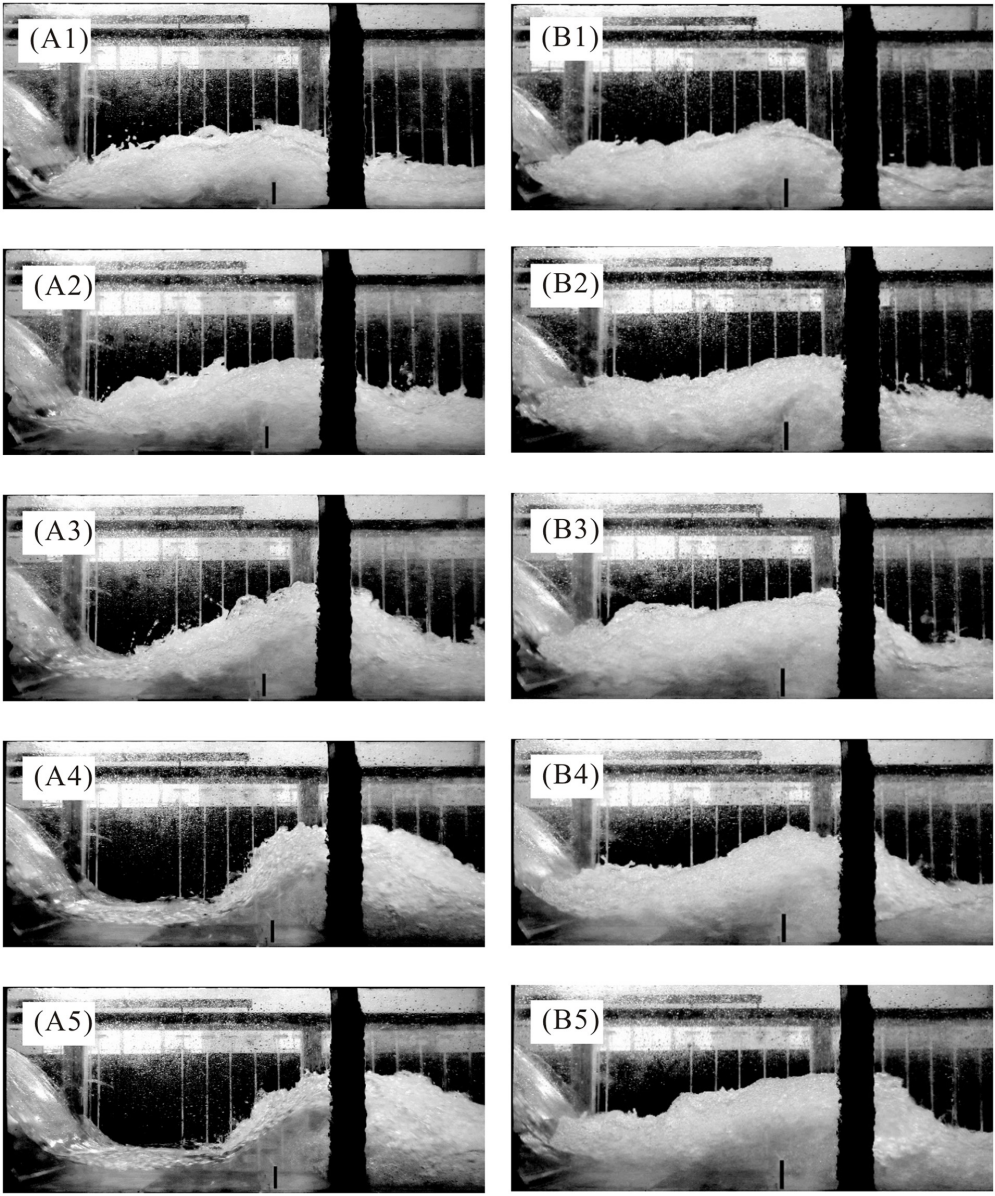
Flow regimes of the PSB for both Case M32 and M33 at different *Fr*_c_. (A) Case M32. (B) Case M33.

Based on preliminary experiments, at low unit discharges, the water surface along the PSB tends to rise, indicating a subcritical flow pattern, due to the sill’s effect. As unit discharge increases, the velocity at the weir toe also increases, and when the unit discharge reaches a certain value, a hydraulic jump occurs due to the flow obstruction caused by the sill. A sill can control the jump and dissipate more energy than a classical hydraulic jump. In this study, the absence of tailwater directly determines the maximum flow depth in the PSB under a consistent discharge. This condition results in varying submergence degrees, thereby influencing the formation of different jump types. Fig 5 illustrates the flow regimes under conditions of large unit discharge, represented in this study by the dimensionless incoming Froude number (*Fr*_c_) at the contraction section, for both Case M32 and M33.

In Fig 5A, with the increase in the unit discharge *q*, *Fr*_c_ decreased from 4.85 to 3.43 and the jump type changed from submerged to repelled jump. Both For *Fr*_c_ = 4.85 and 4.08 (Fig 5(A1) and 5(A2)), it was evident that the hydraulic jump occurred upstream of the contraction section, i.e., the submerged jump. The entire flow became aerated, and there was nearly no clear water near the basin bottom. When *Fr*_c_ was 3.76 by increasing discharges (Fig 5(A3)), the location of jump occurrence slightly moved downstream to the contraction section. The clear water region expanded at the bottom while the area of the roller was reduced. At *Fr*_c_ = 3.61 and 3.43, as seen in Fig 5(A4) and 5(A5), the jump location moved rapidly downstream to the sill’s vicinity. The difficulty of entrapping air bubbles within the lower part of the flow resulted in clear water near the sill. For comparison, In Fig 5B, with a higher sill height for Case M33, the jump type existed in the form of the submerged jump for all approach flow conditions. Although the clear water region also increased as *Fr*_c_ decreased due to the jump roller deflection, the basin still had a more aeration performance than Case M32. Fig 5 also demonstrates that while the jump location varies significantly with the unit discharge based on the flow pattern, especially with the repelled jump approaching closer to the sill, the distance from the highest point of the jump to the sill remains relatively constant.

#### Submergence degree

**Fig 6:**
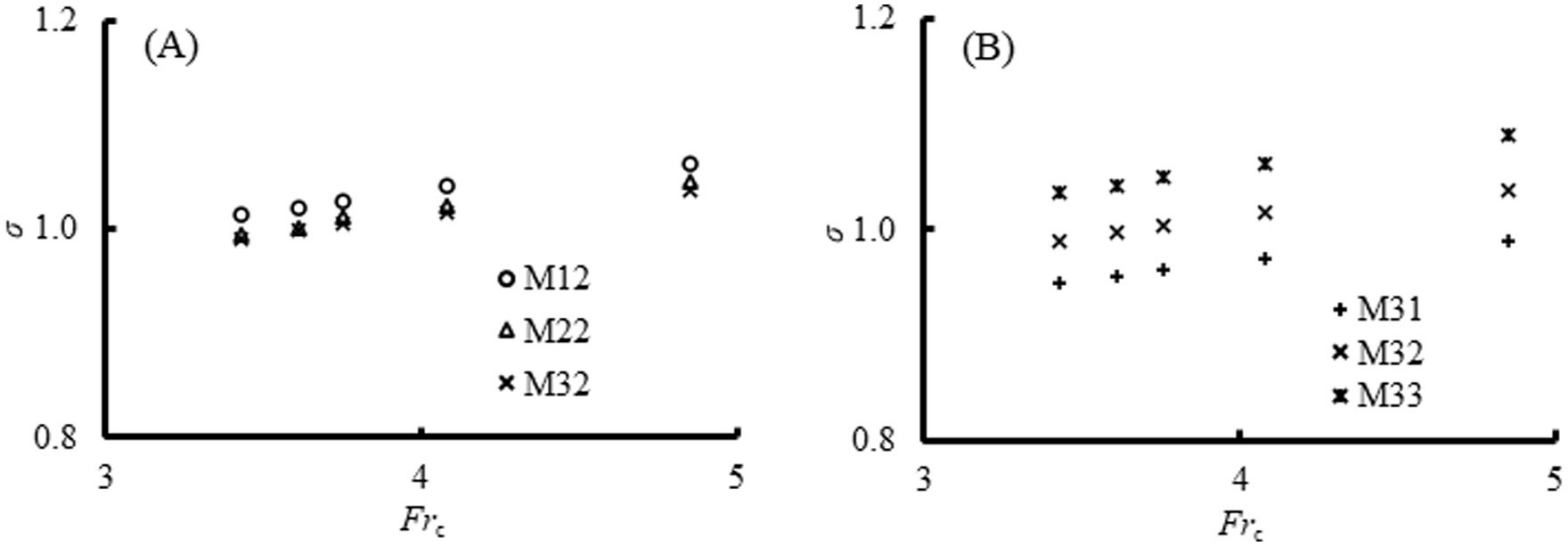
Variations of *σ* with *Fr*_c_ concerning the effect of *l*_b_ and *s*_b_. (A) Effect of *l*_b_. (B) Effect of *s*_b_.

Considering the flow regimes were represented by the submergence degree *σ* and a similar change in flow regimes was also observed in other cases. It was important to investigate the relationship between *σ* and the geometric parameters of the PSB (i.e., the basin length *l*_b_ and sill height *s*_b_). The variation of submergence degree *σ* with the incoming Froude number *Fr*_c_ concerning the effect of *l*_b_ and *s*_b_ is illustrated in Fig 6. In all the operational conditions examined in this study, while different flow patterns correspond to varying values of *σ*, these values predominantly fluctuate around 1.0, with a minimum of 0.95 and a maximum of 1.09. Obviously, both in Fig 6(A) and 6(B), *σ* was in a good linear relationship with *Fr*_c_ no matter what the value of *l*_b_ and *s*_b_ was. In addition, *σ* increased with the decreasing *l*_b_ and increasing *s*_b_.

**Fig 7:**
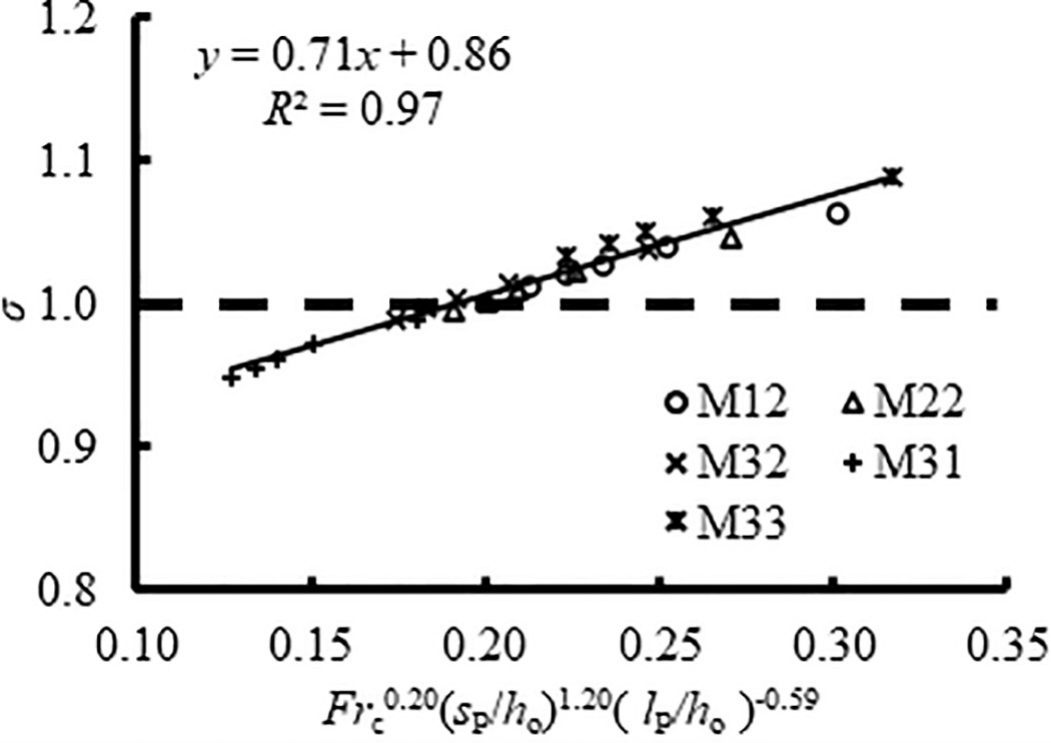
Variation of *σ* with *Fr*c0.2(*s*_b_/*h*_1_)^1.20^(*l*_b_/*h*_1_)^-0.59^.

Fig 7 presents the relationship between *σ* and its influencing factors, and the best fit is,

$$\sigma =0.71Fr_{c}^{0.20}{\left(s_{\text{b}}/h_{1}\right)}^{1.20}{\left(l_{\text{b}}/h_{1}\right)}^{-0.59}+0.86,\;\text{with}\;R^{2}=0.97$$ (8)

where 3.43 ≤ *Fr*_c_ ≤ 4.85, 0.38 ≤ *s*_b_/*h*_1_ ≤ 0.60, and 2.92 ≤ *l*_b_/*h*_1_ ≤ 4.63. Firstly, Eq (8) not only demonstrated the relationship between the submergence degree and dimensionless length and sill height of the PSB (i.e., *σ* increased with increasing *Fr*_c_ and *s*_b_/*h*_1_ but decreased with increasing *l*_b_/*h*_1_) but also the magnitude of the effect of these parameters. The sill height appeared to have more effect on changing the flow regime than *l*_b_ and *Fr*_c_. From Case M22 and M32 in Fig 6, it was also evident that the influence of *l*_b_ on *σ* is minimal as the free jump is approached. Secondly, the thick dash line (*σ* = 1) in Fig 7 expressed the free jump, which resulted in *Fr*_c_^0.2^(*s*_b_/*h*_1_)^1.20^(*l*_b_/*h*_1_)^-0.59^ ≈ 0.2. Accordingly, the part above the line (*σ* > 1) represented the submerged jump for *Fr*_c_^0.2^(*s*_b_/*h*_1_)^1.20^(*l*_b_/*h*_1_)^-0.59^ > 0.2 and the part below (*σ* < 1) stood for the repelled jump for *Fr*_c_^0.2^(*s*_b_/*h*_1_)^1.20^(*l*_b_/*h*_1_)^-0.59^ < 0.2. It was efficient to control the flow regimes by altering the length and sill height of the PSB for a given *Fr*_c_. In practical applications, both the outflow aeration effect and energy dissipation are closely linked to the flow regime, specifically the submergence degree. By examining the inflow conditions, sill height, and sill position, an initial prediction of the flow regime can be made, aiding in the optimization the PSB design.

### Outflow aeration effect

#### Outflow air concentration at the sill position

**Fig 8:**
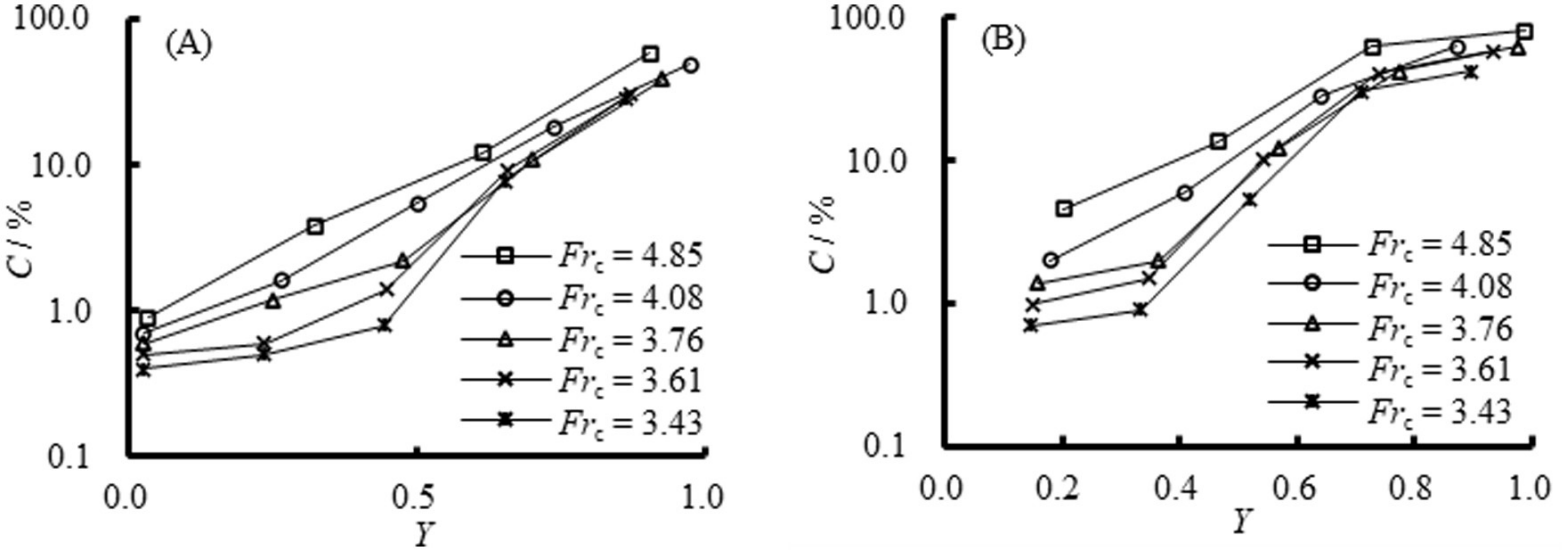
Air concentration at the sill position. (A) Case M32. (B) Case M33.

Fig 8 shows air concentration (*C*) at the sill position under different *Fr*_c_ for both Case M32 and M33, corresponding to the flow regimes as shown in Fig 5. In Fig 8, the air concentration *C* is expressed in logarithmic form, and *Y* = (*y*–*s*_b_)/(*y*_max_−*s*_b_), represents the relative measurement position to the flow depth above the sill, where *y* is the height from the basin bottom to the air concentration measurement point, and *y*_max_ is the height from the basin bottom to the flow surface. Both in Fig 8A and 8B, the air concentration *C* increased along the flow depth at the sill position, and the value increased rapidly especially when *Fr*_c_ = 4.08 and 4.85. But for *Fr*_c_ = 3.76, 3.61, and 3.43, there were sharp values in the vicinity of *Y* = 0.5 for M32 and *Y* = 0.36 for M33, respectively. It was revealed that the air concentrations at the sill position for a higher sill height increased more rapidly, as associated with the observation of submerged jump for M33 at relatively large discharges. Secondly, in Fig 8A, *C* decreased as *Fr*_c_ decreased, which was related to the transformation of the submerged jump into the repelled jump. In Fig 8B, *C* also exhibited a decrease when *Fr*_c_ continued to decrease, although the flow regime was always a submerge jump. Furthermore, the air concentration *C* for M33 was slightly larger than the value measured in M32 under identical *Fr*_c_, which again illustrated that a larger air concentration at the sill position was achieved with a higher sill height. In summary, the air concentration at the sill position was closely correlated with flow regimes under identical approach flow conditions.

#### Outflow air concentration downstream of the sill

Based on the design purpose, the use of an PSB could be considered to obtain a pre-aerated flow. The measured air concentration data (i.e., the outflow air concentration of the PSB) downstream of the sill were also analyzed to justify the aeration effect and cavitation potential of the outflow. Peterka [1] proved that the cavitation damage could be considerably reduced when the air concentration on the structure surface was 1.0–2.0%. For this type of pre-aerated stilling basin spillway (PSBS) with sidewall constrain, it is desirable that the air concentration both near the bottom and the sidewall reaches at least 1%.

**Fig 9:**
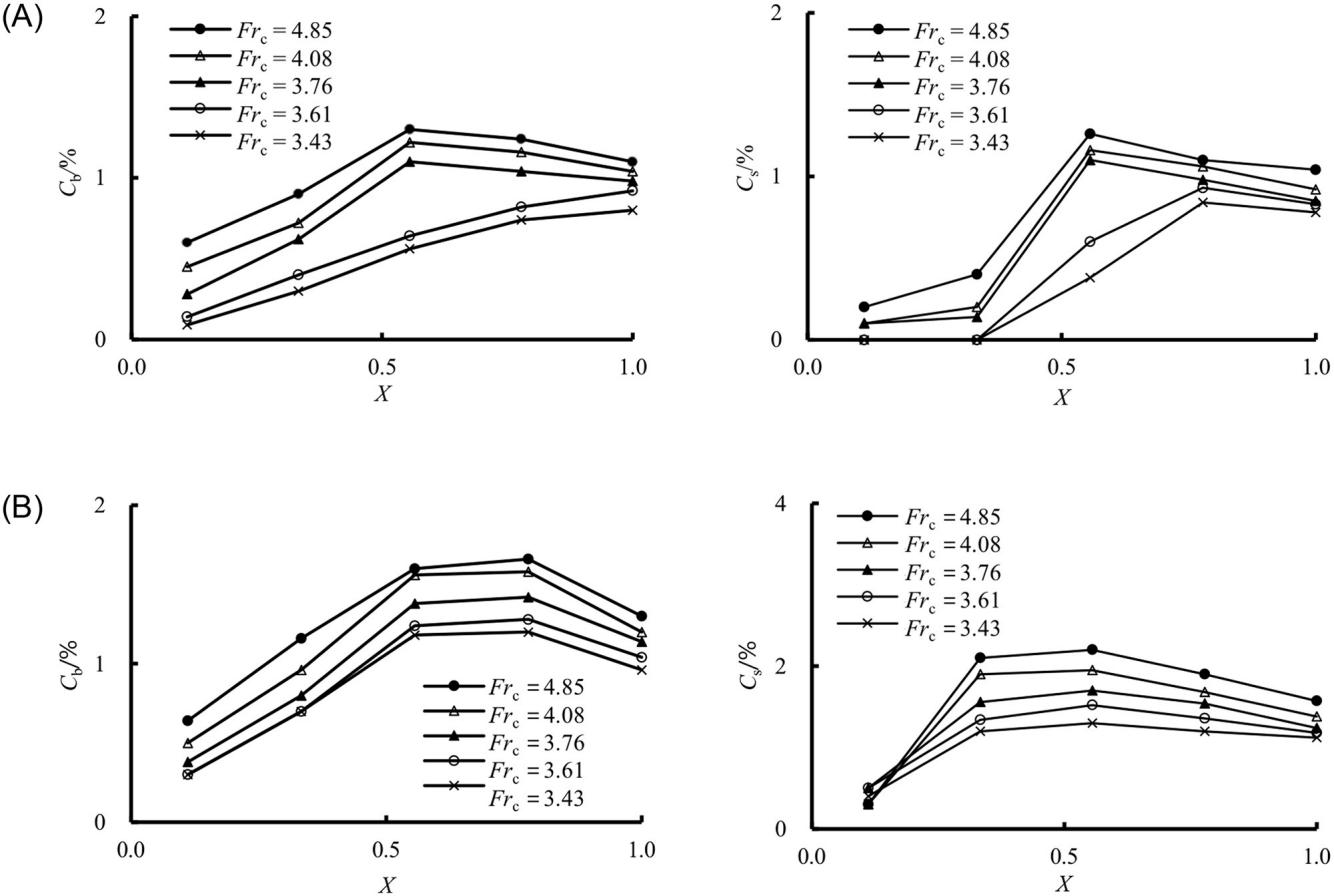
Variation of *C*_b_ and *C*_s_ downstream of the sill under all *Fr*_c_. (A) Case M32. (B) Case M33.

Fig 9 shows the variation of air concentration both on the bottom *C*_b_ and sidewall *C*_s_ downstream of the sill at all *Fr*_c_ for both Case M32 and M33. In this figure, *X* = (*x*-*l*_b_)/(*x*_max_-*l*_b_) represents the location of the measurement point relative to the length of the measurement region, where *x* is the length from the measurement point to the sill position, *x*_max_ is the length from the measurement point to the last measurement point. Considering solely from the perspective of the pre-aeration design, the length of the stilling basin should generally not be excessively prolonged. In all the schemes discussed in this study, the last measuring point is established at two times the length of the stilling basin downstream, namely 0 < *X* ≤ 1.

From Fig 9, regarding M32 (Fig 9A) and M33 (Fig 9B), despite the variation in the jump initiation location caused by the sill height differences (as depicted in Fig 5), the air concentrations *C*_b_ and *C*_s_ both initially escalated and then progressively diminished with the increase in distance *X*. Combining the flow patterns, it can be observed that due to the obstruction of the sill, the aerated water flow cannot reach the rear part of the sill, resulting in very small air concentration in this area. Subsequently, the aerated water flow causes a rapid increase in air concentration within a certain range behind the sill. However, beyond a certain range, the air concentration decreases due to deaeration. Considering the characteristics of hydraulic jump, the location of the deaeration is downstream of the jump impact. Despite the decrease in air concentrations *C*_b_ and *C*_s_, the rate of augmentation in air concentration was more pronounced than the subsequent decrease. The air concentrations both at the bottom and sidewall could still reached minimum value of 1.0% and 1.2%, respectively, even at *X* = 1 from the PSB. Heller [29] and Hohermuth et al. [30] noted that small-scale models tend to underestimate the rate of air entrainment due to scale effects, with prototype air concentrations consistently higher than those measured in the models. Additionally, the impact of different scales on air entrainment may vary. The air concentration downstream of the sill was effective in reducing cavitation damage. Secondly, consistent with the air concentration at the sill position, there is an apparent trend of decreasing air concentration downstream of the PSB as the incoming Froude number (*Fr*_c_) increased. And the value of the air concentration was also very sensitive to the sill position and sill height. For example, as depicted in Fig 9B, an increase in sill height from 6.5 cm to 8.0 cm yielded a discernible difference in air concentrations (*C*_b_ and *C*_s_) compared to Fig 9A.

**Fig 10:**
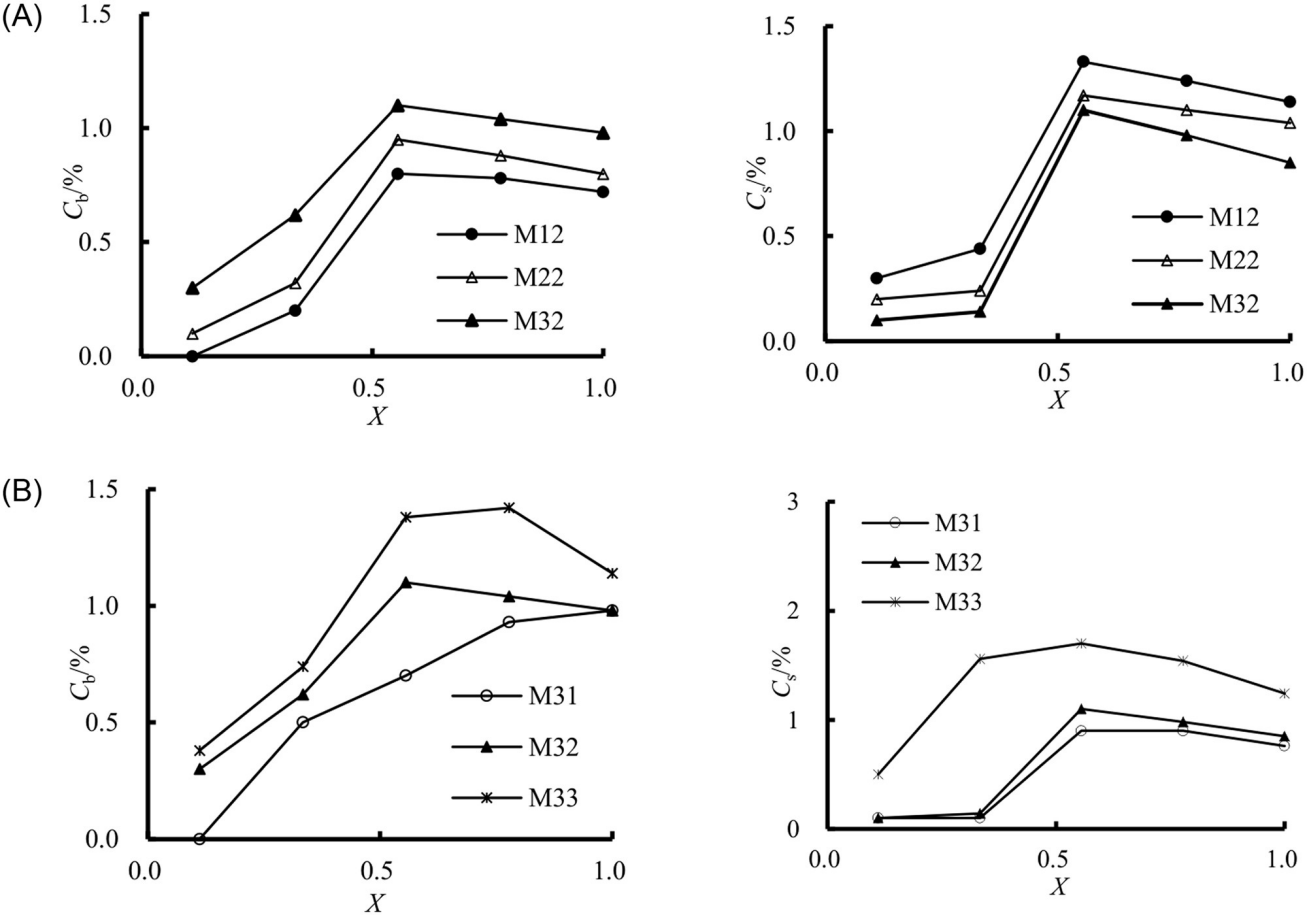
Variation of *C*_b_ and *C*_s_ concerning the effect of *l*_b_ and *s*_b_ at *Fr*_c_ = 3.43. (A) Effect of *l*_b_. (B) Effect of *s*_b_.

Furthermore, the variation of air concentration on the bottom and sidewall downstream of the sill concerning the effect of the length and sill height of the PSB at the same incoming Froude number (*Fr*_c_ = 3.43) is shown in Fig 10. It was noted that *C*_b_ at the same position was associated with an increase in the length of the PSB. In contrast, the shorter length had slightly higher *C*_s_ for *s*_b_ = 0.065 m. Under the condition of *l*_b_ = 0.7 m, increasing the sill height could effectively increase the air concentration both on the bottom and sidewall. In summary, the air concentration was intimately related to the flow regime, irrespective of the inflow conditions or the sill configuration. Because of the air concentration influenced by the scale effect, the outflow air concentration of the PSB (both at and downstream of the sill position) in the prototype should be further modified in the future.

Overall, the sill for the traditional stilling basins is typically positioned near a location corresponding to the length of classical hydraulic jump at the designed discharge. In this study, the sill in the PSB is placed closer to the weir. At this point, changes in the sill’s position and height significantly impact the outflow aeration effect. It can be observed that when *X* = 0.56 to 0.78, the maximum aeration concentration is achieved. Additionally, the position and height of the sill affect water surface fluctuations near the sill caused by the jump, which in turn impacts the transition of the water flow into the downstream spillway. Taking all these factors into account, determining the sill’s position and height is crucial for the entire structure (pre-aerated stilling basin spillway). This should be a primary focus of future research on the entire structure.

### Energy dissipation

Although the primary purpose of the PSB design is pre-aeration, it also possesses energy dissipation characteristics due to its deviation from the Type I stilling basin (with a significantly shortened length compared to the Type I stilling basin, except for the sill configuration). This effectively reduces the flow velocity in the transition section of spillway. This paper compares the relative local energy loss (Δ*H*/*h*_1_) of the PSB and another stilling basin characterized by an abrupt bottom rise [31], i.e., an positive step, under varying inflow conditions. Disregarding the influence of the sill and step configuration and considering only the inflow Froude number (*Fr*_c_), the relationship between Δ*H*/*h*_1_ and *Fr*_c_ for these two structures can be determined as follows:

$$\frac{\Delta H}{h_{1}}=0.551Fr_{c}^{2},\;\text{for}\;\text{a}\;\text{sill}\;\text{with}\;R^{2}=0.68$$ (9)

$$\frac{\Delta H}{h_{1}}=0.629Fr_{c}^{2},\;\text{for}\;\text{a}\;\text{positive}\;\text{step}\;\text{with}\;R^{2}=0.92$$ (10)

As illustrated in Eqs (9) and (10), the relative local energy loss Δ*H*/*h*_1_ for a sill exhibits a larger deviation than for a positive step with the coefficient of determination *R*^2^ decreasing from 0.92 to 0.68. Compared to a sill, a positive step can be regarded as an infinitely long sill, rendering the influence of its length negligible. When considering both the inflow conditions and the height of the sill, the relationship between Δ*H*/*h*_1_ and *Fr*_c_ and *s*_b_/*h*_1_ can be derived as follows:

$$\frac{\Delta H}{h_{1}}=0.086Fr_{c}^{2.689}{\left(s_{\text{b}}/h_{1}\right)}^{0.390},\;\text{for}\;\text{a}\;\text{sill}\;\text{with}\;R^{2}=0.87$$ (11)

$$\frac{\Delta H}{h_{1}}=0.135Fr_{c}^{2.462}{\left(s_{b}/h_{1}\right)}^{0.288},\;\text{for}\;\text{a}\;\text{positive}\;\text{step}\;\text{with}\;R^{2}=0.99$$ (12)

**Fig 11:**
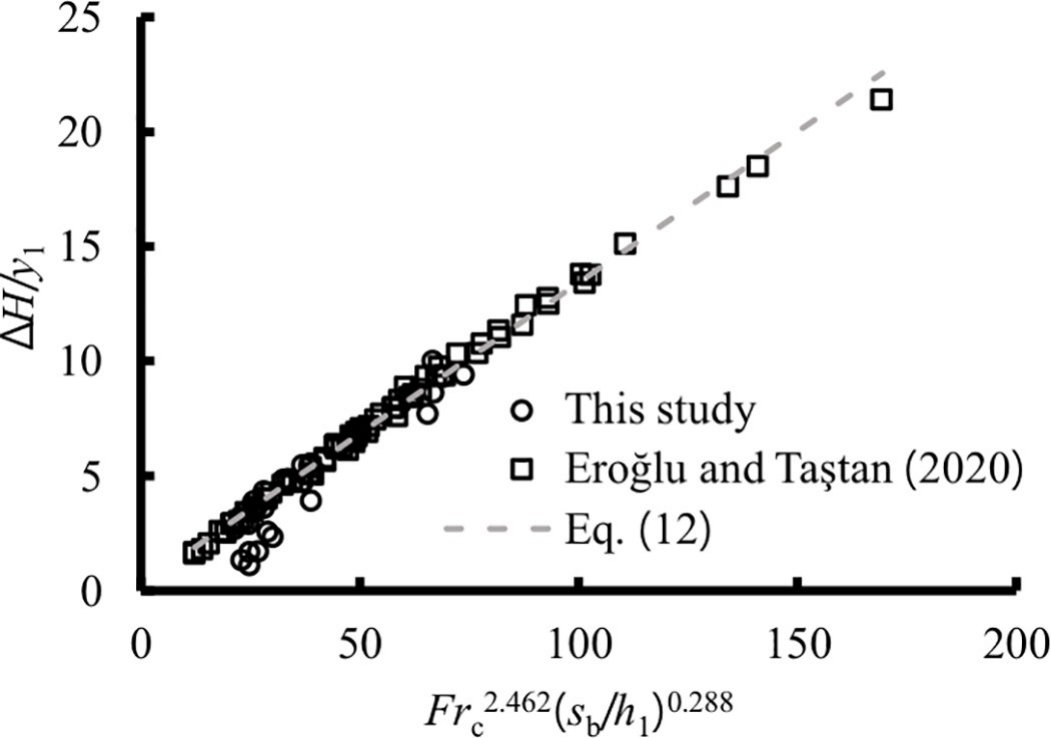
Estimations of local relative local energy loss.

From Eqs (11) and (12), it was observed that the fitting accuracy of the estimated relative local energy loss is higher when both the height of the sill or step and inflow conditions are considered, compared to when only the inflow conditions are considered. This difference is particularly evident for a sill configuration between Eqs (9) and (11). The graph of Eq (12) is presented in Fig 11. Notably, under the same inflow conditions and sill or step height, the relative local energy loss for a positive step is greater.

Further analyzing the impact of the sill position on energy dissipation, and integrating the influence of the three main factors discussed in the dimensionless analysis, the formula for relative local energy loss is derived as follows:

$$\frac{\Delta H}{h_{1}}=0.08Fr_{c}^{3.285}{\left(l_{b}/h_{1}\right)}^{-0.318}{\left(s_{\text{b}}/h_{1}\right)}^{0.430},\;R^{2}=0.90$$ (13)

It can be observed that, in the context of a sill positioned very close to the weir, (i.e., the PSB), both the height and location of the sill significantly impact energy dissipation. Similar to the flow pattern characterized by submergence degree *σ*, the sill height influences energy dissipation comparably to its position. Overall, through the study of inflow conditions and their combined effect with the sill configuration, it is evident that considering only the inflow conditions shows a larger deviation. The energy dissipation effect of the PSB can be effectively predicted based on both inflow conditions and sill height. This is crucial for analyzing the impact of pre-aeration on the energy dissipation in the entire structure (i.e., the pre-aerated stilling basin spillway). Furthermore, the analysis highlights that the sill configuration has a significantly impact on the relative local energy loss Δ*H*/*h*_1_. The primary factor causing differences is the flow pattern; in the formation of various jump types (i.e., free, repelled, and submerged jump), the sill configuration plays a crucial role. Additionally, the impact of the jump on the PSB bottom after passing the sill is considerable. It was evident that without the downstream impact, the direct entry into the downstream spillway requires further attention.

## Conclusion

In the present work, a novel structure called a pre-aerated stilling basin (PSB) was introduced and used to prevent cavitation damage inflicted by the absence of air entrainment along the spillway. Concerning this new structure, the PSB played a role in pre-aerating the flow through a hydraulic jump. Based on the analysis of flow regimes and outflow aeration effect of the PSB, the main findings are summarized as follows:

1. The jump type changed from the submerged jump to the repelled jump with the decrease of the incoming Froude number at the contraction section. Associated with various jump types, the submergence degrees increased with the decreasing length and increasing sill height of the PSB. The sill height appeared to have more effect on changing the flow regime than the sill position and the incoming Froude number.
2. The air concentration at and downstream of the sill position in the PSB was significantly influenced by the flow regime, sill height, and sill length, showing an initial increase and then a gradual decrease in concentration with distance. This variation was crucial for reducing cavitation damage, and adjustments may be needed in prototype applications due to scale effects.
3. For PSB energy dissipation, considering only inflow conditions results in larger deviations, highlighting the significant impact of sill configuration on relative local energy loss. The primary factor causing these differences is the flow pattern, with the sill configuration playing a crucial role in the formation of various jump types.
4. Generally, considering the further development of aerated flows in the downstream spillway, as long as the outflow from the basin was sufficiently aerated, the downstream air concentration along the spillway tended to be substantial. Future research should focus on the aeration basin, such as contemplating alterations to the form of the sill configuration. Additionally, it is imperative to examine the impact of the actual conjunction with the spillway on its aeration.

## Supporting information

S1 Appendix Air entrainment data. (XLSX)

S2 Appendix Energy dissipation data. (XLSX)

S3 Appendix Submergence degree data. (XLSX)
